# Advanced maternal age alters expression of maternal effect genes that are essential for human oocyte quality

**DOI:** 10.18632/aging.102864

**Published:** 2020-02-25

**Authors:** Jing-Jing Zhang, Xiaoyan Liu, Li Chen, Shouxin Zhang, Xia Zhang, Cuifang Hao, Yi-Liang Miao

**Affiliations:** 1 Institute of Stem Cell and Regenerative Biology, College of Animal Science and Veterinary Medicine, Huazhong Agricultural University, Wuhan 430070, Hubei, China; 2 Key Laboratory of Agricultural Animal Genetics, Breeding and Reproduction (Huazhong Agricultural University), Ministry of Education, Wuhan 430070, Hubei, China; 3 Reproductive Medicine Centre, Affiliated Hospital of Qingdao Medical University, Yuhuangding Hospital of Yantai, Yantai 264000, Shandong, China; 4 National Demonstration Center for Experimental Veterinary Medicine Education, Huazhong Agricultural University, Wuhan 430070, China

**Keywords:** ovary aging, oocyte, human, *TOP2B*, scRNA-seq

## Abstract

To investigate the effects of maternal age on the quality of oocytes, we used single-cell RNA sequencing to detect global gene transcriptome and identify key genes affected by advanced age in human mature oocytes. We isolated mRNA from mature oocytes obtained from IVF or ICSI patients (three oocytes from younger (≤30 years) and three oocytes from older (≥40 years) patients for scRNA-seq. We identified 357 genes differentially expressed between matured oocytes from older and younger women's. The up-regulated genes were significantly enriched with annotations related to transcriptional activation, oxidative stress and immune function, while down-regulated genes were enriched with catalytic activity. The key candidate gene *TOP2B* was found by protein interaction network analysis, and knockdown verification on younger mouse matured oocytes showed that *TOP2B* was a key gene affecting the oocyte quality and early embryo development. These results will contribute new knowledge on the molecular mechanisms of female ovary aging and establish a criterion to evaluate the quality of oocytes in women with advanced maternal age.

## INTRODUCTION

It is well known that oocyte quality determines the fertilized embryo’s developmental potential. The developmental potential of oocytes declines with increasing age in all species and this phenomenon is termed ovary aging. In humans, the predominant feature of female ovary aging is that the number and quality of oocytes decline. Human ovary aging begins at the age of 30 and lasts until menopause [1]. Increasingly, in our modern society, women are postponing childbearing and with increasing age, the risk of infertility, abortion and birth defects is increased accordingly [2]. It has now become an important social concern that ovary aging leads to a decline in fertility. Therefore, it is necessary to reveal the underlying molecular mechanisms of ovary aging and establish a criterion to evaluate the quality of oocytes in women with advanced maternal age.

The main factors affecting the fertility of older women include decreased uterine implantation ability, oocyte and embryos quality. However, studies have shown that uterine implantation capacity and follicular loss in older women are not associated with infertility. These findings strongly suggest that oocyte quality is the major factor in the fertility decline in older females. Clinical data from Assisted Reproductive Technology (ART) cycles further support this conclusion. It is very difficult to achieve live birth for older women after transferring their own fertilized oocytes; however, if donor oocytes from young healthy individuals are used, the ovarian aging effects can be reduced [3]. Therefore, the biological age of the oocyte determines the quality of the oocyte and significantly affects the fertility outcome.

As shown in Supplementary Table 1, we have summarized the general differences between younger and older oocytes. Since human oocyte chromosome aneuploidy increases with age, most reproductive aging studies emphasize the importance of chromosomal abnormalities in reducing the potential for female gamete development [4–7]. Subsequently, other age-related nuclear morphological changes, such as abnormal protein expression associated with spindle assembly checkpoints in older oocytes, were found to result in incorrect chromosome segregation leading to oocyte aneuploidy [8–12]. Since telomeres can mediate aging of mitotic cells, older oocytes may be derived from precursors that have undergone more DNA replication cycles, suggesting that abnormal meiosis in older women may be due to telomere shortening [13]. The function of mitochondria affects the quality of oocytes and contributes to fertilization and embryo development [14]. Older oocytes show mitochondrial DNA damage, abnormal mitochondrial gene expression, and decreased mitochondrial membrane potential [15–19]. A recent study showed that the nucleoli of oocytes from reproductive old mice have relatively prominent fibril centers and dense fibril centers, and the cytoplasm contains more ribosomes [20]. In addition, genome-wide microarray analysis of human MII oocytes has demonstrated age-related differences in transcript abundance [21], while the age of women had no effects on gene expression profiles of human germinal vesicle (GV) oocytes [22]. Therefore, we selected oocytes from the MII phase as the research object to understand the molecular mechanism of the decline in oocyte quality in older women.

Compared to microarray analysis, the information from single-cell RNA sequencing (scRNA-seq) analysis is more accurate. The scRNA-seq technology can be used to reveal molecular events related to oocyte maturation and early embryonic development. In our study, we employed the scRNA-seq technique to compare the transcriptome of matured *in vivo* oocytes from younger and older women to identify differences in intracellular stored mRNA and indicate the molecular mechanisms of female ovary aging and establish a criterion to evaluate the quality of oocytes in women with advanced maternal age.

## RESULTS

### Female fertility declines with advanced reproductive age

**Figure 1:**
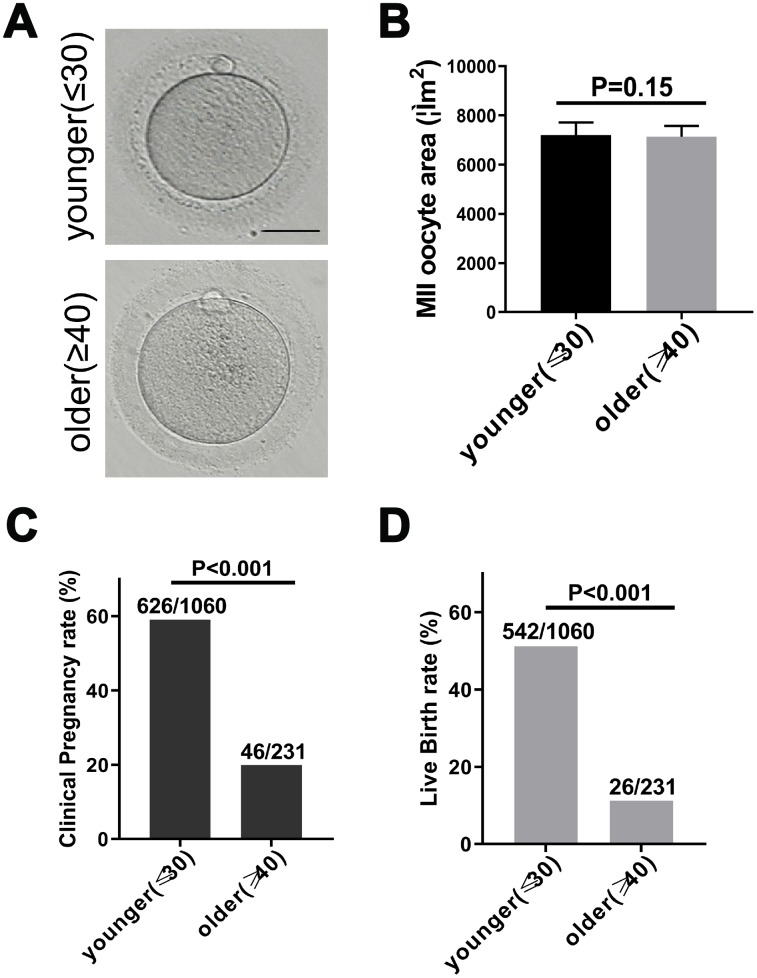
**Fertility declines in women with advanced reproductive age.** (**A**) The morphology of matured oocyte from a younger and an older female patient. Bars = 50μm. (**B**) Younger (27.6 ± 1.3 years, n = 170) and older women (41.6 ± 1.8 years, n = 183) MII oocyte area, without zona pellucida. Unpaired two-tailed t-test. (**C**) Statistics of clinical pregnancy rate during the 2014-2017 fresh embryo transfer cycle. Relative Risk (RR) estimates used to compare probabilities. RR = 0.17, 95% CI = 0.12 – 0.24, P < 0.001. (**D**) Statistics of live birth rate during the 2014-2017 fresh embryo transfer cycle cycle. RR = 0.12, 95% CI = 0.08 – 0.18, P < 0.001.

To investigate whether maternal age affected the quality of matured human oocytes, we examined the morphology of human MII oocytes from younger (≤30 years old) and older (≥40 years) patients under the microscope. The perivitelline space of the two groups of MII oocytes was same, the polar body was intact, and the oocyte morphology was the same (Figure 1A). In mice, oocyte diameters have been detected as a function of age and indicate oocyte quality [20]. Therefore, we calculated the area of younger women (27.6 ± 1.3 years, n = 170) and older women (41.6 ± 1.8 years, n = 183) MII oocytes. There was no significant difference in the area of human oocytes (Figure 1B). Next, we separately calculated the clinical pregnancy rate and live birth rate of younger and older women in the 2014-2017 fresh embryo transfer cycle. 19.91% (46/231) of female patients of advanced age had a clinical pregnancy, while 59.06% (626/1060) of female patients of younger age had a clinical pregnancy. This indicated a 83% reduction in the likelihood of clinical pregnancy in older patients (RR: 0.17 [0.12, 0.24]; p < 0.001) (Figure 1C). Similarly, 11.25% (26/231) of older patients experienced a live birth, while 51.13% (542/1060) of younger women had a live birth. This indicated an 88% reduction in the likelihood of a live birth in older patients (RR: 0.12 [0.08, 0.18]; p < 0.001) (Figure 1D). These results indicate that the clinical pregnancy and birth rates of younger and older women are significantly reduced with age.

### Globally expressed genes of single human MII oocytes

**Figure 2:**
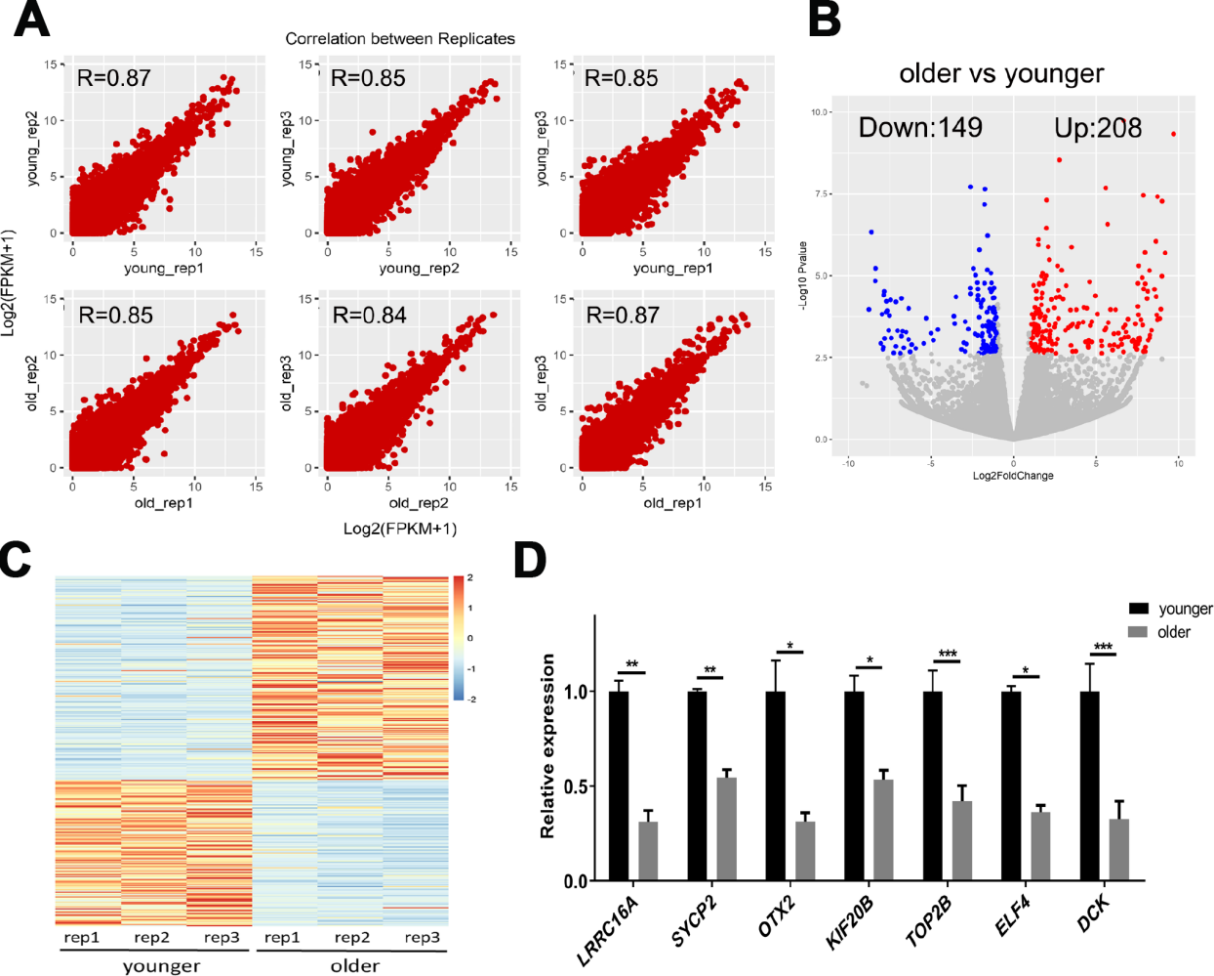
**scRNA-seq of younger and older matured human oocytes.** (**A**) Scatter plot compares the amount of scRNA-seq gene expression (FPKM) between different biological replicates. The spearman correlation coefficients are shown. (**B**) DEGs between oocytes of younger (27.0 ± 1.0 years, n = 3) and older women (43.3 ± 2 years, n = 3) are shown in the volcano map. Genes that express higher (up-regulated) in older female oocytes are shown in red, and genes that are lower (down-regulated) are shown in blue. (**C**) The gene expression profile heat map divided 357 DEGs into two groups, with up-regulated expression (red) and down-regulated expression (blue) in older women. The color corresponds to the z-score per gene calculated from FPKM. (**D**) A total of 7 DEGs were selected for qRT-PCR validation, unpaired two-tailed t-test.

To determine differences in transcript abundance between younger and older oocytes, we performed a scRNA-seq on three younger (27.0 ± 1.0 years) and three older (43.3 ± 2.1 years) female MII oocytes using the Smart-seq2 protocol. We obtained 21–35 million monoclonal reads uniquely mapped to the genome for each sample (Supplementary Table 2). The spearman correlation coefficient between biological replicates was greater than 0.80, indicating that the biological repeat of scRNA-seq exhibited highly reproducible results (Figure 2A). Then, we identified 357 genes in younger and older patients with more reliable differential expression in oocytes (Figure 2B, 2C and Supplementary Data 1). We further selected the seven DEGs for qRT-PCR verification. *SYCP2*, *KIF20B* and *LRRC16A* are genes related to chromosome separation, cytokinesis and the cytoskeleton. *ELF4* initiates translational switching by interaction with the RINGO binding complex. *OTX2* and *DCK* are randomly selected DEGs. *TOP2B* is related to chromatin structure and gene expression regulation. The expression of selected genes in the qRT-PCR analysis was consistent with the fold change in transcriptome analysis (Figure 2D), which validated the accuracy and reliability of the sequencing data.

### GO term and KEGG pathway enrichment analyses of the DEGs

**Figure 3:**
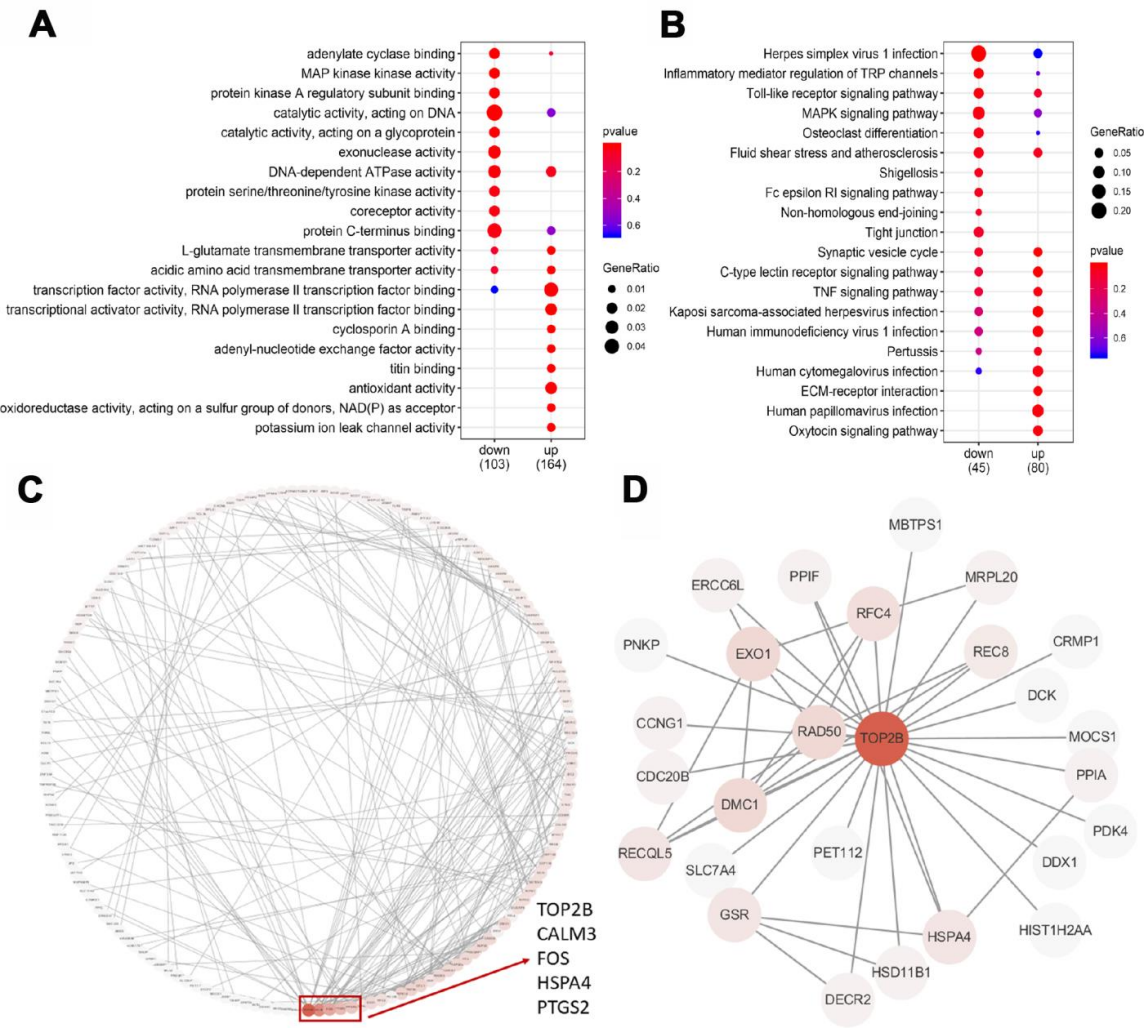
**GO, KEGG and PPI analysis of DEGs.** (**A**) The top 20 GO terms were presented in the enrichment analyses of DEGs within the older oocytes. (**B**) The top 20 KEGG pathways were presented in the enrichment analyses of DEGs within the older oocytes. (**C**) Using the STRING online database, a total of 181 DEGs were filtered into the DEGs PPI network complex. The red box encloses a gene with more than 10 proteins interacting with the protein. (**D**) PPI subnetworks of TOP2B.

To test which biological processes are involved in the 357 DEGs, we enriched the up-regulated and down-regulated genes into the GO terminology and the KEGG pathway, respectively. For up-regulated genes, the most enriched GO terms are related to transcriptional activation, transmembrane transport, oxidative stress and titin binding (Figure 3A). Briefly, two GO terms were associated with transcriptional activation, including transcriptional activator activity (p=0.003) and transcription factor activity (p=0.009). The enrichment of 3 GO terms for transmembrane transport was adenyl-nucleotide exchange factor activity (p=0.005), L-glutamate transmembrane transporter activity (p=0.007), acidic amino acid transmembrane (p=0.010) and potassium ion leak channel activity (p=0.003). The enrichment of 2 GO terms for oxidative stress was antioxidant activity (p=0.008) and oxidoreductase activity (p=0.010). For down-regulated genes, the most enriched GO term is catalytic activity (Figure 3A). Briefly, eight GO terms were associated with catalytic activity, including adenylate cyclase binding (p=0.003), protein kinase A regulatory subunit binding (p=0.005), MAP kinase activity (p=0.003), catalytic activity acting on DNA (p=0.006), catalytic activity acting on a glycoprotein (p=0.008), exonuclease activity (p=0.011), DNA-dependent ATPase activity (p=0.013) and protein serine/threonine/tyrosine kinase activity (p=0.024). These results strongly suggested that age-related decline in oocyte quality included complicated and multifactorial regulation.

The KEGG database was used to refine the potential signaling pathways in our data. Regardless of whether the gene is up-regulated or down-regulated, the most abundant KEGG pathway is immune function (Figure 3B). The enrichment of 11 KEGG pathways of immune function is the C-type lectin receptor signaling pathway (p=0.004), Kaposi sarcoma-associated herpesvirus infection (p=0.012), human papillomavirus infection (p=0.019), human immunodeficiency virus 1 infection (p=0.020), TNF signaling pathway (p=0.026), human cytomegalovirus infection (p=0.027), Pertussis (p=0.042), Herpes simplex virus 1 infection (p=0.002), Inflammatory mediator regulation of TRP channels (p=0.020), Toll-like receptor signaling pathway (p=0.021), Fluid shear stress and atherosclerosis (p=0.045).

### *TOP2B* is a key gene for the decline of oocyte quality in advanced age

To identify the key candidate genes, we used the STRING online database (online: http://string-db.org) and Cytoscape software to analyze protein interaction network. A total of 181 DEGs of the 357 commonly altered DEGs were filtered into the DEGs PPI network complex, containing 181 nodes and 267 edges, and 176 of the 357 DEGs did not fall into the DEGs PPI network. Among the 417 nodes, 4 central node genes were identified with the filtering of degree >10 criteria (i.e., each node had more than 10 connections), and the most significant 30 node degree genes were *TOP2B, CALM3, FOS,* *HSPA4* and *PTGS2* (Figure 3C). In addition, *TOP2B* was down-regulated in older oocytes, and we extracted the PPI network of *TOP2B* and its interacting proteins (Figure 3D). Previous reports demonstrated that changes in chromosome-related proteins (such as cohesions and kinetochore proteins) might cause chromosome segregation errors with increasing age, while *TOP2B* interacted with cohesins and CTCF at topological domain boundaries [23, 24]. Therefore, *TOP2B* was selected as a key candidate gene.

### Depletion of *TOP2B* in mouse MII oocytes led to early embryo arrest at the 2-cell stage

**Figure 4:**
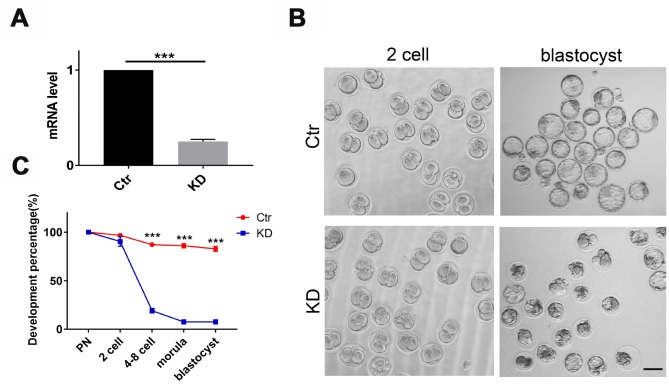
**Depletion of *TOP2B* in mouse matured oocytes led to early embryo arrest at the 2-cell stage.** (**A**) *TOP2B* knockdown efficiency in young mouse MII oocytes was assessed by qRT-PCR. Paired two-tailed Student’s t-test. (**B**) Representative images of mouse early embryos knocking down *TOP2B* mRNA. Bars = 100μm. (**C**) Knocking down *TOP2B* significantly reduces the developmental rate of early embryos (n=35,35,35). Paired two-tailed Student’s t-test.

To test whether *TOP2B* was a key gene responsible for the quality decline in older oocytes, we knocked down *TOP2B* in matured oocytes of young mice to mimic the decrease in *TOP2B* expression in matured oocytes of older women. First, we verified knockdown efficiency by quantifying *TOP2B* mRNA levels, with knockdown efficiency around 70% (Figure 4A). Next, we observed early embryonic development at PN, 2-cell, 4-8 cell, morula and blastocyst stages after IVF. The embryo morphology of the knockdown group and the control group was similar at the 2-cell stages; however, most control embryos developed into blastocysts while most of the *TOP2B* knock-down embryos were arrested at the 2-cell stage (Figure 4B). The embryo developmental rate was counted for each time period, and the embryo developmental rate was significantly reduced in the knockdown group after the 2-cell stage (Figure 4C). In conclusion, these results indicate that mouse early embryos are arrested at the 2-cell stage if *TOP2B* mRNA levels were decreased in MII oocytes.

## DISCUSSION

In this study, we firstly compared the transcriptional profiles of MII oocytes in younger (27.0 ± 1.0 years, n=3) and older (43.3 ± 2 years, n=3) women by scRNA-seq and identified 357 differently expressed genes related to maternal age in human MII oocytes. Among them, genes up-regulated in older MII oocytes are mainly enriched with transcriptional activation, oxidative stress, titin binding and immune function, while genes down-regulated are mainly enriched with catalytic activity.

Intracellular stored mRNAs in matured oocytes are transcribed from the maternal genome during oogenesis and important for the early embryo development after fertilization [25, 26]. We found that transcriptional activators are up-regulated in MII oocytes in older women, including *FOS, PPARA, THRA, NOTCH2, ANKRD1, TBX20,* and *NR2E3* encoding transcription factors. Changes of these transcription factors indicate that the global transcriptional activity in the oocytes may be significantly dysregulated with increased oocyte age.

Consistent with previous studies, DNA damage and reproductive oxidative stress increased with reproductive aging [13], which is partly responsible for the lower developmental capacity of aged oocytes. Previous studies had found that oocytes from older women had reduced DNA damage repair due to lower efficacy of DNA double-strand breaks repair mechanisms and shorter telomeres [27]. Consistently, our data also found that *RAD50* and *RAD17* were down-regulated in oocytes of older women, resulting in a reduced ability of oocyte DNA damage repair.

Interestingly, we found that 11 down-regulated genes in older MII oocytes were involved in the catalytic enzyme activity, including *AKAP6, AKAP5, MAP2K6, MAPK10, TOP2B, DDX1, RAD50, EXO1, ERCC6L, FUT8,* and *GCNT3*. Among them, *TOP2B* was confirmed to be down-regulated in older oocytes by qRT-PCR (Figure 2D). The nuclease *TOP2B* had the highest frequency of protein-protein interactions with other proteins (Figure 3C). These results were consistent with previous studies showing that *TOP2B* was less active in senescent neurons [28]. In addition, these fertilized oocytes were arrested at the 2 cell stage after we knocked down *TOP2B* in young mouse MII oocytes (Figure 4B). On one hand, *TOP2B* regulated and altered the topological state of DNA, promoting double-strand break repair after oxidative stress [29, 30]; on the other hand, *TOP2B* interacted with cohesins and CTCF [23], thereby promoting rapid expression of genes in early embryo development.

The differentially expressed genes found in this study and the genes found in the Grondahl et al study showed a 10% (27/258) overlap. Compared to the study by Barragán et al, our study has only one of the same DEG (*ANXA5*) that is up-regulated in the oocyte of older patients. Combined the two studies of Grondahl and Barragán, we found that more genes’ expression might be affected by age when women are older than 40 years of age [21, 31]. Grondahl et al. divided two groups (age <36 years and age=37-39 years) and Barragán et al. divided groups of 20-35 year-olds. This might be the reason that there were more than 90% differential genes in our studies that could not overlap with Grondahl and Barragán’s results.

In summary, there is a significant difference in gene expression profiles between younger and older MII human oocytes. Our findings are important to identify the molecular mechanisms of ovarian aging in women, and establish a criterion to evaluate the quality of oocytes in women preparing for pregnancy.

## MATERIALS AND METHODS

Women who were infertile for non-ovarian reasons (such as tubal blockage, uterine disease and male infertility) in the Reproductive Medicine Centre of Yuhuangding Hospital of Yantai were included in this study. The women had no other diseases which may affect ovarian function, such as polycystic ovarian syndrome, diabetes, and other endocrine and metabolic diseases. All patients were younger than 30 years old or older than 40 years old. Each female patient provided one oocyte, and normal sperm was selected for *in vitro* fertilization.

### Human matured oocyte collection

All women were stimulated with follitropin alpha (Hemeiqi®, Ferring, Germany), with daily injections of 150–300 IU. Pituitary suppression was performed with a GnRH antagonist (0.05 mg of Cetrorelix acetate, Tianxing®, Chengdu Tiantaishan Pharmaceutical Co., Ltd, China) administered daily from day 16 of stimulation. When three or more follicles of ≥18 mm of diameter were observed, final oocyte maturation was triggered with a dose of 6000-10000 IU of the human chorionic gonadotrophin (hCG, Livzon Pharmaceutical Group Inc, China). Oocyte collection was performed 36 hr later by means of ultrasound-guided transvaginal follicular aspiration.

Individual MII human oocytes were exposed to hyaluronidase (80 units/ml; Sigma-Aldrich, USA) and manipulated with a Stripper (Origio, USA) tip to remove all remaining cumulus cells, and then washed three times in Dulbecco's phosphate buffered saline with 0.01% polyvinylpyrrolidone (Sigma-Aldrich, USA). Samples were stored in PicoPure RNA extraction buffer (10 μl), heated to 42°C for 30 min, and subsequently stored at −80°C until total RNA purification as per manufacturer's protocol (Thermo Fisher Scientific, USA).

### RNA sequencing of single-cell transcriptome libraries and analysis

The sample in the single oocyte collection tube was directly amplified by the Smart-Seq2 method [32]. The libraries were sequenced by an Illumina Hiseq X-ten platform with 150 bp paired-end. Reads of RNA-seq were mapped to the human genome (GRCh38) with STAR (v2.5.3a) [33]. Mapped reads were subsequently assembled into transcripts guided by the GENCODE reference annotation with Stringtie (v1.3.3b) [34]. Expression level of each gene was quantified with normalized fragments per kilobase of exon per million reads mapped (FPKM). Gene differential expression analysis using DESeq2 (v1.20.0) [35] based on the reads count file obtained by HTSeq (v0.9.1) [36]. Spearman’s r coefficient was calculated using the core function with default parameters. Gene ontology (GO) analysis and Kyoto Encyclopedia of Genes and Genomes (KEGG) pathway analysis was performed using clusterProfiler R package (v3.8) [37]. The differential genes were imported into the STRING database to obtain the interaction relationship between the differential genes, and the interaction network map was drawn using Cytoscape (v3.6.1) [38].

### qRT-PCR

scRNA-seq validation was performed by qRT-PCR analysis using 5 ng (in triplicates) of the cDNA libraries previously constructed. qRT-PCR was performed with SYBR Premix Ex Taq (Takara, Kusatsu, Japan) in a reaction volume of 10 μl and the Bio-Rad CFX Connect system (Bio-Rad, Hercules, USA). Delta-delta Ct value represented the mRNA expression, and the data were normalized to the amount of *ACTB* expressed. Primers were listed: *ACTB* (F: CAGAAGGAGATCACTGCCCTGG/R: ACTCCTGCTTGCTGATCCACAT); *DCK* (F: GCCAAAGCCTTGAATTGGATGGA/R: GCCTTGCTCTTCATTTCTTCCCC); *IGFBP6* (F: CAATTCTGCGGGTGTCCAAGAC/R: ACGTAGAGTGTTTGAGCCCCTC); *EIF4G2* (F: ACAAGTCAGTGCCCTGTATGCT/R: GCCTTGCTCTTCATTTCTTCCCC); *KIF20B* (F: TTCTTTACGGAGTCAGGCATCCA/R: TTGGCTCGTTTTGGTTGAGACAC); *LRRC16A* (F: ATCTAGCCCGAAAGTTGCCCTT/R: GCCATCTTTACTGGAGGACCGT); *OTX2* (F: GACCCGGTACCCAGACATCTTC/ R: GCGGCACTTAGCTCTTCGATTC); *SYCP2* (F: GCGAACAACAGAGGCTTCATCT/R: GCCTGTCTTGCAGCACTTTCAT); *TOP2B* (F: GGCGATTATAACCCTGGCAGGA/R: AGAGAAGGTGGCTCAGTAGGGA).

### Mouse matured oocyte collection, IVF and embryo culture

Female mice were super-ovulated by using 5 IU equine CG followed 48 h later with 5 IU human CG (hCG). MII oocytes were collected 13–14 h of hCG administration into Whitten’s-Hepes medium containing 0.01% polyvinyl alcohol (Whitten’s- Hepes-PVA), and cumulus cells were removed using 0.1% hyaluronidase. For IVF, epididymal sperm from a CD-1 male was collected into 500 μL of HTF medium and allowed to capacitate for 1.5–2 hr before use. Oocytes were inseminated for 60 min in a 100 μL drop of HTF/BSA containing 2 × 10^6^ sperm per mL, and then the oocytes were quickly washed through three drops of Ca^2+^-free CZB medium by using a thin bore pipette to remove unbound sperm. Embryos were cultured in KSOM medium (EMD Millipore, MR-106-D) in a humidified atmosphere of 5% CO_2_.

### siRNA interference

Mouse MII oocytes were microinjected with 5–10 pl of non-targeting or *TOP2B*-targeting siRNA (Genepharma, Shanghai, China) in Hepes-buffered Whitten medium. The working concentration of siRNA was 25 μM. TOP2B siRNA sequences: TOP2B-MUS-1303:5’-GCA GCUAUGUAGACCUUUATT-3’; TOP2B-MUS-2898: 5’-GCUGCAAGCCCUCGUUAUATT-3’; TOP2B-MU S-2565:5’-GCAACAAAGCAUUUGACAUTT-3’.

### Statistical analysis

All percentages from at least three repeated experiments were expressed as mean ± SD. Statistical analyses were implemented with R (http://www.r-project.org/). Clinical pregnancy rates and live birth rates were calculated and tested for differences using a log binomial model. Student’s t-test was used to compare means using the t.test function. In the graphed data *, **, and *** denote *P*-values of <0.05, 0.01 and 0.001, respectively.

### Ethics approval

All the procedures in the present study were reviewed, supported and approved by the Ethic Committee of Life Science of Yuhuangding Hospital of Yantai (2016-177) and the Animal Care and Use Committee of Huazhong Agriculture University. All experiments were performed in accordance with relevant guidelines and regulations of the committees. We obtained agreement, permission and signed consent from all patients included in the present study.

## Supplementary Material

Supplementary Tables

Supplementary Data
