# Supplementary material for: Advanced maternal age alters expression of maternal effect genes that are essential for human oocyte quality
Source: Aging (Albany NY). 2020 Feb 25;12(4):3950–61. doi: 10.18632/aging.102864 (PMC7066876; doi:10.18632/aging.102864)
Supplement: Supplementary Tables [file aging-12-102864-s001..pdf]

SUPPLEMENTARY TABLES

Supplementary Table 1. The general differences between younger and older oocytes.

	Young oocytes	Aging oocytes	Reference
Frequency of chromosomal abnormalities	Low	High	[4–7]
Spindle checkpoint regulation	Proper	Abnormal expression of proteins associated with spindle assembly checkpoints leads to incorrect chromosome segregation	[8–12]
Telomeres	Intact	Telomere shortening and decreased telomerase activity	[13]
Mitochondrial function	Normal	mtDNA damage, changes in mitochondrial gene expression and kinetics, and a decrease in mitochondrial membrane potential	[15–19]
Nucleolar structure and number of ribosomes	Nucleolar structure is intact, the number of ribosomes is proper.	Nucleolus has a relatively prominent fibril center and dense fibril center, cytoplasm contains more ribosomes	[20]

Supplementary Table 2. The mapping information for scRNA-seq samples.

Sample	Layout	Total reads	Data size	Q30 bases ratio	Uniquely mapped reads	Uniquely mapped ratio	Multiple mapped reads	Multiple mapped ratio
younger-rep1	PE150	24000444	7.20G	0.94	22725781	0.95	775650	0.03
younger-rep2	PE150	36978423	11.09G	0.92	34774163	0.94	1165577	0.03
younger-rep3	PE150	30211047	9.06G	0.95	28272099	0.94	1351522	0.04
older-rep1	PE150	24752022	7.43G	0.94	23563150	0.95	815626	0.03
older-rep2	PE150	23300590	6.99G	0.94	21978244	0.94	1006665	0.04
older-rep3	PE150	23978243	7.19G	0.94	22584550	0.94	960795	0.04
